# In Situ Polymer Gel Electrolyte in Boosting Scalable Fibre Lithium Battery Applications

**DOI:** 10.1007/s40820-024-01451-z

**Published:** 2024-06-28

**Authors:** Jie Luo, Qichong Zhang

**Affiliations:** Key Laboratory of Multifunctional Nanomaterials and Smart Systems, Chinese Academy of Sciences, Suzhou Institute of Nano-Tech and Nano-Bionics, 398 Ruoshui Road, Suzhou, 215123 People’s Republic of China

**Keywords:** High-performance fibre lithium batteries, Gel electrolytes, Channel structures, Stable interface, Scalable application

## Abstract

The poor interfacial stability not only deteriorates fibre lithium-ion batteries (FLBs) performance but also impacts their scalable applications. To efficiently address these challenges, Prof. Huisheng Peng team proposed a generalized channel structures strategy with optimized in situ polymerization technology in their recent study. The resultant FLBs can be woven into different-sized powering textiles, providing a high energy density output of 128 Wh kg^-1^ and simultaneously demonstrating good durability even under harsh conditions. Such a promising strategy expands the horizon in developing FLB with particular polymer gel electrolytes, and significantly ever-deepening understanding of the scaled wearable energy textile system toward a sustainable future.

## Highlights

- Stable interfaces were successfully achieved through designing channel structures in electrodes to sufficiently incorporate polymer gel electrolyte fabricated through in situ polymerization.
- The resultant fibre lithium battery (FLB) demonstrated superior energy density output of 128 Wh kg^−1^ and enabled scalable production capability.
- Such high-performance FLBs presented prospect applications in diverse scenarios, for example, firefighting, space exploration, and human–computer interaction, even under harsh environments.

## Graphical abstract

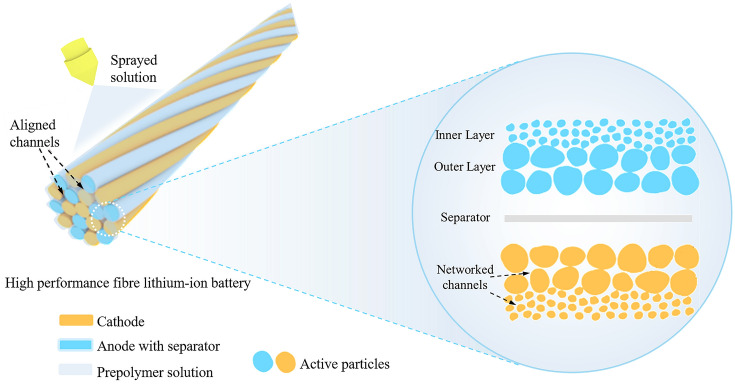

The flourishing development of electronic textiles has prompted increasingly diverse wearable applications, and urgent demands of corresponding energy storage devices [1]. It's noted that the conventional rigid, bulky planar powering system greatly affects their air permeability, light weight, and real applications. Consequently, it is crucial to develop novel high-performance flexible energy storage devices. Fibre lithium-ion batteries (FLBs) worked as ideal power solutions recently arose of significant interest [2]. The known liquid-electrolyte fibre energy storage devices have high ionic conductivity and favorable electrode/electrolyte interfaces. However, they may suffer from leakage, flammability, and hermetic sealing safety worries, which boosts the pursuit of an alternative replacement. Comparatively, semi-solid electrolytes are safer and demonstrate higher flexibility and mechanical strength than liquid electrolytes. Given these strengths, Lu et al. proposed a promising hierarchical channel strategy for constructing industrially producible fibre batteries with optimized in-situ polymerization technology, which allows the formation of intimate interface compatibility and improves ion transport [3].

Unlike liquid or pure solid electrolytes, polymer gel electrolytes possess superior advantages in terms of establishing a stronger interface with electrodes, exhibiting both the diffusive characteristics of liquids and the mechanical properties of solids [4]. These enable FLBs can be woven into flexible power textiles with large-scale production. Lu et al. rotated multiple cathode fibres and anode fibres with separators together to form aligned channels, in which the fibre electrodes deposited initially small and then large active particles. The sprayed high liquidity acrylate-based monomer solution with low viscosity endows sufficient infiltration capability in the pores of the aligned channels, networked inner small and large channels within seconds, simultaneously following thermal induced polymerization to prepare gel electrolyte (Fig. 1a). The successful combination of aligned channels and networked channels was essential for FLBs with sufficient infiltrated gel electrolytes. In particular, the aligned channels showed lower polarizations and higher double-layer capacitances, obtaining a considerable specific capacity output. The outer large networked channels are responsible for both high Coulombic efficiencies and high specific capacity outputs, while the inner tiny dense channels provide closer contacts with the current collectors and form rich lithium-ion migration routes, which are conducive for more Li^+^ ions intercalation. As a consequence, it reduced the internal resistance three times and increased the discharge capacity almost five times than that of the conventional solution casting method. Moreover, such hierarchical channel constructions are capable of reducing the negative impacts of volume variations during the charge/discharge process. There are no obvious interface detachments occurring between the electrodes and gel electrolyte even at the harsh temperature range of − 40 ~ 80 °C or after 100,000 cycles mechanical deformations treatments, such as bending, twisting, stretching, etc. As such, the fibre electrodes were fabricated by a confined deposition and can be efficiently rotated together with gel electrolyte, presenting a full industrial scalability. Taking the rapid ions migration and specific capacity into consideration, a multi-axis rotating method comprising of optimal six fibre electrodes was applied to achieve continuous production with a rate of 3,600 m h^−1^ per winding unit. It is obviously higher than the solution-extrusion method [5]. The output specific capacity of the resultant FLBs reached 128 Wh kg^−1^, which is much higher than that of other reported methods with liquid electrolytes (Fig. 1b, c). Additionally, the resulting FLBs maintain excellent safety and overall performance after long electrochemical cycles.

**Fig. 1:**
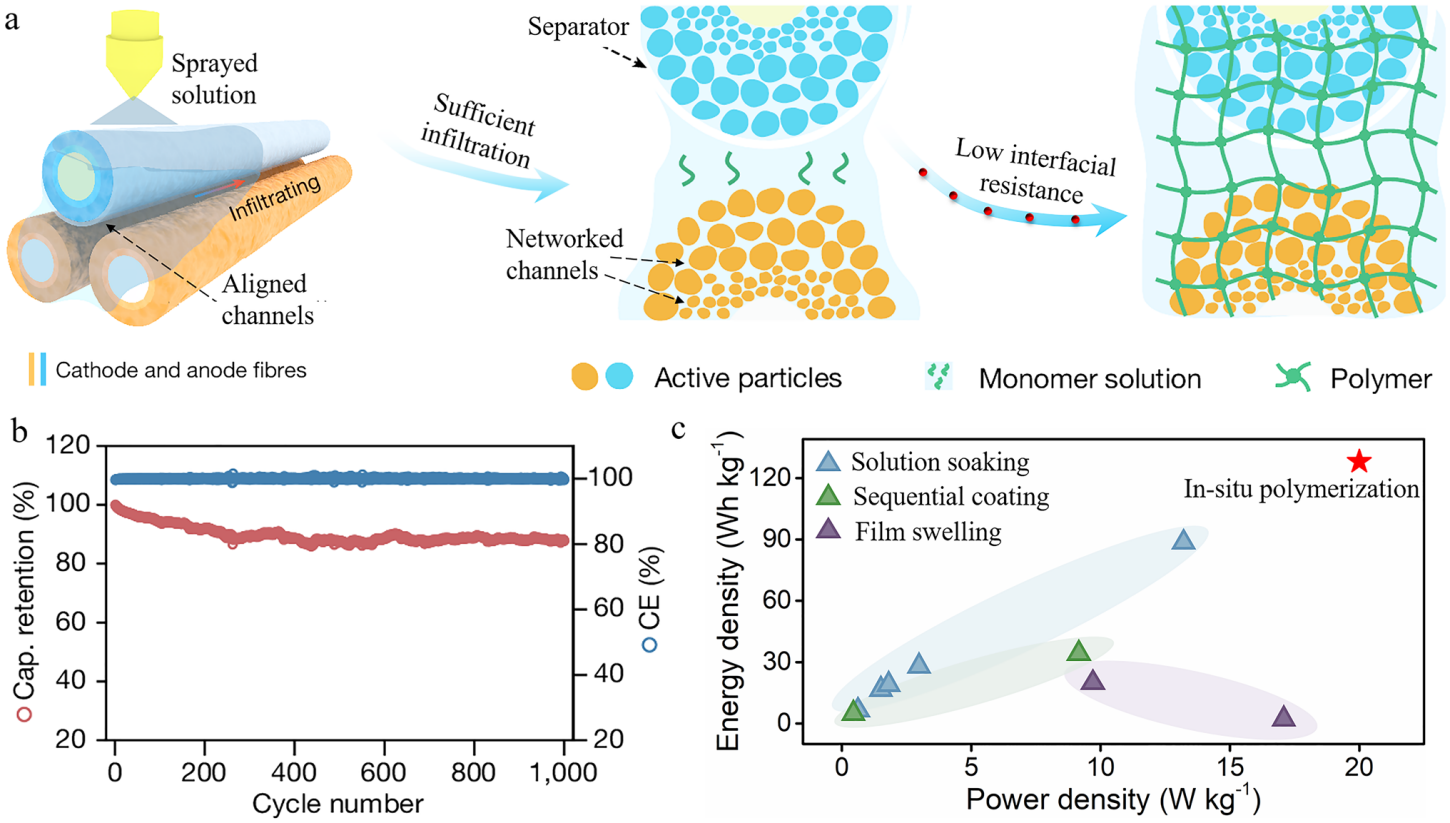
Channel structures design assisted in situ polymerization strategy for improving the interfacial stability and electrochemical performance of FLBs. **a** Fabrication of an FLB with polymer gel electrolyte. **b** The electrochemical properties of FLBs with polymer gel electrolyte. **c** The electrochemical properties of FLBs compared with other previous electrolytes. Adapted from Ref. [3]. Copyright©2024, The Author(s), under exclusive licence to Springer Nature Limited

Contributing to these advantages, various monomers and corresponding energy storage devices were also fabricated by taking advantage of this versatile method, further certificating this generalized strategy. Thus, Lu et al. woven the FLBs into 50 cm × 30 cm flexible power textiles and demonstrated a capacity of around 3,000 mAh, a power density of 1.48 mW cm^−2^ at 600 mA, demonstrating certain competitiveness against those commercial batteries. The voltage plateau preserves above 4.29 V even after standing for 400 h at different temperatures or vacuum conditions. Impressively, the durable FLB textiles concurrently resist washing and friction treatments with negligible capacity loss as well as high safety even if they are suffering immersion in ice, boiling water, or subjected to cut off. Remarkably, this consecutive production realized a big size textile with 30 m × 0.3 m, building an impressive trade-off in terms of high stability and real applications, especially in the fields of firefighting suits and space exploration. After integrating the FLBs into firefighting suits for powering temperature and gas sensors, the textiles could determine a wide temperature range of 26 ~ 116 °C and harmful gas concentration with low limits, prominently eliminating the liquid electrolyte leakage and flaming safety issues. Another potential application was serving space exploration. It exhibited a perfect powering capability for a light board under ultraviolet light and − 40 °C. The successful application demonstrations of the fabricated FLB textiles strongly confirmed the rational channel designs and the importance of polymer gel electrolytes for high-performance FLBs.

Although FLBs have witnessed giant achievements, the choice of gel polymer electrolytes in developing desired battery performance, environmental sustainability, and device assembly should also be emphasized scientifically [6]. On this basis, several deeper outlooks of advancing the electrolyte were proposed and discussed in detail: (i) in-depth study of gel electrolyte. As a generalized process technology, the on-site thermally initiated polymerization with azo initiator abandons the complex processes (solution extrusion, thermal drawing, continuous coating, and electrospinning method, etc.), distinctly reducing production costs. However, the issues of the polymerization mechanisms, monomer utilization, generated voids, swelling, decomposition, and residuals remain to be overcome, otherwise deteriorating battery performance. Not only the main formulation needs meticulous optimization,but the configuration designs of polymer chains and gel electrolytes are also carefully considered. A thorough understanding of these aspects can pave the way for guiding the design of polymer gel electrolytes with rapid charge transfer, less side reactions, high stability, and enhanced performance; (ii) machine learning-assisted design of polymer gel electrolytes. Even though significant experimental progresses have been made, the FLB with polymer gel electrolyte is a complicated system and need versatile prediction tools, like deep learning algorithms,and artificial intelligence, to explore optimal battery performance and corresponding gel electrolyte. Cui group successfully utilized a machine learning model to identify key features for achieving a remarkable performance electrolyte [7]. Such advancements can effectively reduce the time and costs, leading to more efficient and durable energy storage systems; (iii) the balance of environmental and performance. The trade-off between environmental sustainability and performance is favourable for sustainable cycling. Searching for more biodegradable electrolytes is a promising approach to address environmental concerns. Moreover, other effort like the recycling strategies is essential as well, which will minimize the waste and environmental pollution, pushing forward a greener future for energy storage.

In addition, the superior polymer gel electrolyte induces FLBs infinite possibilities when used as the wearable power source. It can be assembled with various sensor devices or wearable electronics. The integration requirements of voltage or power consumption matching, external circuits design, wireless communication protocols, hardware connection interfaces, and so on should be precisely tailored. All the above efforts will contribute to more sustainable wearable battery technologies and an environment-friendly future.
